# Putting the Spotlight Back Onto the Flanker Task in Autism: Autistic Adults Show Increased Interference from Foils Compared with Non-autistic Adults

**DOI:** 10.5334/joc.369

**Published:** 2024-05-23

**Authors:** Daniel Poole, James A. Grange, Elizabeth Milne

**Affiliations:** 1 Department of Psychology, University of Sheffield, UK; 2 School of Psychology, Keele University, UK

**Keywords:** selective attention, autism, flanker task, drift diffusion model

## Abstract

Autistic people may have a less focused spotlight of spatial selective attention than non-autistic people, meaning that distracting stimuli are less effectively suppressed. Previous studies using the flanker task have supported this suggestion with observations of increased congruency effects in autistic participants. However, findings across studies have been mixed, mainly based on research in children and on response time measures, which may be influenced by differences in response strategy between autistic and non-autistic people rather than differences in selective attention.

In this pre-registered study, 153 autistic and 147 non-autistic adults completed an online flanker task. The aims of this study were to test whether increased congruency effects replicate in autistic adults and to extend previous work by fitting a computational model of spatial selective attention on the flanker task to the data. Congruency effects were increased in the autistic group. The modelling revealed that the interference time from the foils was increased in the autistic group. This suggests that the activation of the foils was increased, meaning suppression was less effective for autistic participants. There were also differences in non-interference parameters between the groups. The estimate of response caution was increased in the autistic group and the estimate of perceptual efficiency was decreased.

Together these findings suggest inefficient suppression, response strategy and perceptual processing all contribute to differences in performance on the flanker task between autistic and non-autistic people.

## Introduction

Autism is a prevalent neurotype in which sensory and perceptual differences are a key feature (Kern et al, 2006; Kirby et al, 2022). Experimental work has highlighted differences in how autistic people selectively attend to stimuli (for reviews see Allen & Courchesne, 2001; Ames & Fletcher-Watson, 2010; Keehn, Müller & Townsend, 2013). For instance, autistic samples show superior performance in visual search tasks (Constable et al, 2020) and process extraneous stimuli under increased perceptual load (Remington, Swettenham, Campbell, & Coleman, 2009; Remington, Swettenham & Lavie, 2012), but are slower at disengaging and shifting attention to peripheral stimuli (Landry & Bryson, 2004; Keehn, Kadlaskar, McNally & Francis, 2019). Autistic people may also suppress non-target stimuli less effectively than non-autistic people, arising from issues with inhibitory control (Geurts, van den Bergh & Ruzzano, 2014; Tonizzi, Giofrè, & Usai, 2022) including an attentional lens (or spotlight) which is less efficient compared with non-autistic people (Burack, 1994). In the current study we compared the performance of autistic and non-autistic adults on a measure of spatial selective attention, the flanker task (Eriksen & Eriksen, 1974), and fitted participants’ data to a model of attentional selection (the shrinking spotlight model; White et al, 2011).

### Flanker Task

The flanker task (Eriksen & Eriksen, 1974) requires participants to make a speeded discrimination judgement about a central stimulus (the target) flanked by other stimuli (foils). The foils can be congruent or incongruent with the central target. Response times are increased and accuracy reduced in the incongruent condition relative to the congruent condition (Eriksen, 1995). This *congruency effect* is used as a measure of the interference from the foils and is a robust effect, with good test-retest reliability (Wöstmann et al, 2013; Zwaann et al, 2018).

Effective performance on the task has been conceptualised by the function of the ‘spotlight’ of spatial selective attention (Eriksen & St James, 1986). The spotlight focuses on the target across the course of the trial, such that the early activation of foils is reduced over time as they are suppressed (Ridderinkhof, 2002). This has been supported by distributional analysis combining response times and accuracy data (Gratton, Coles & Donchin, 1992). Conditional accuracy functions have been used to plot accuracy in each condition by quantiles of the response time distribution. Typically, the error congruency effect (difference in accuracy rate between the conditions) is largest in the fastest response time quantiles but diminishes for slower responses (e.g. Stins et al, 2007; Hübner & Töbel, 2019). That is, participants make errors on incongruent trials when responding quickly, whereas when taking more time to respond the accuracy rate is comparable to that on congruent trials. The shrinking spotlight model (SSP; White, Ratcliffe & Starns, 2011, White et al, 2018) provides a theoretical account of the temporal dynamics of spatial selective attention on the flanker task.

**Figure 1:**
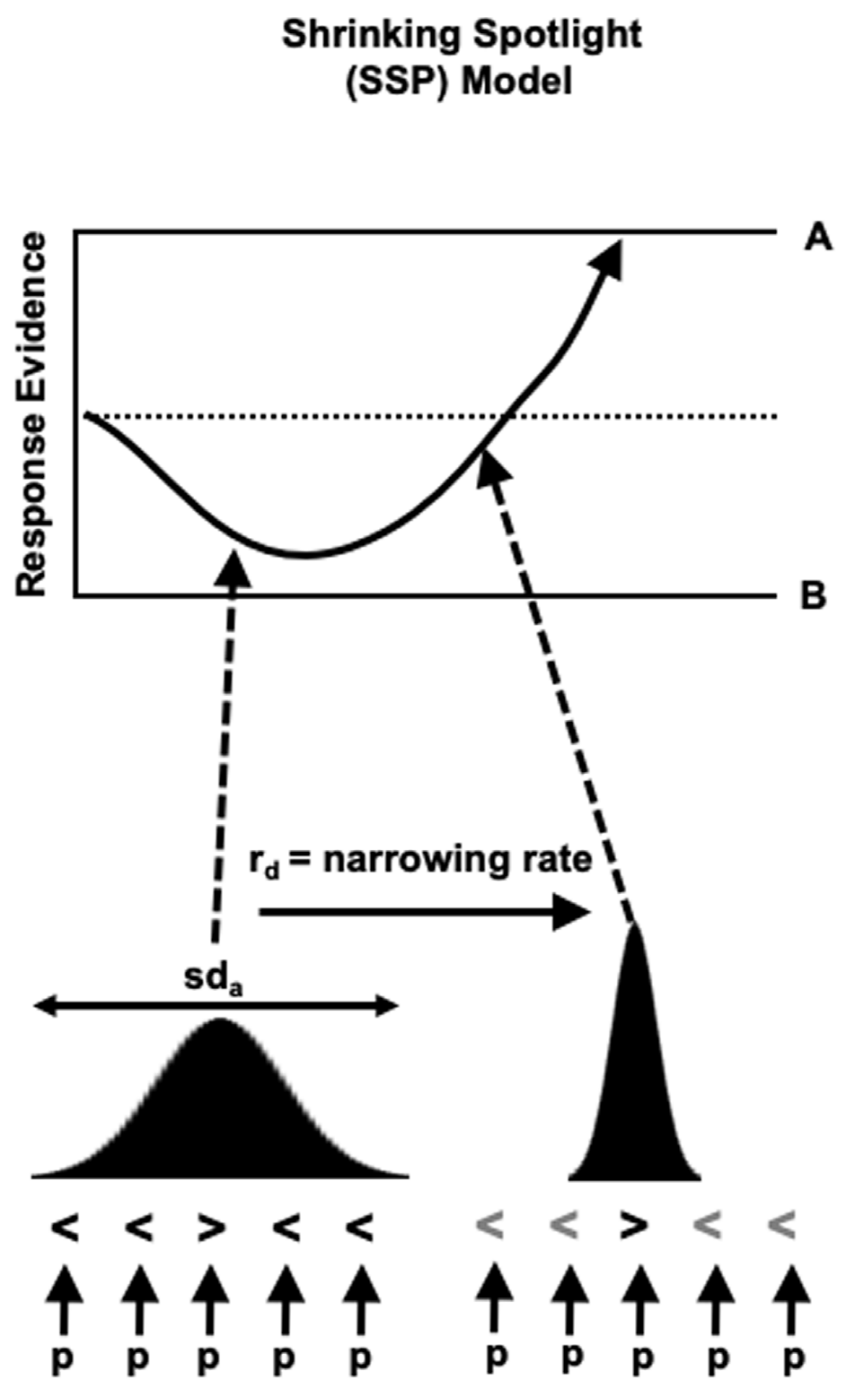
Schematic of response selection process on an incongruent trial on the flanker task according to the Shrinking Spotlight Model (SSP). The drift rate of the evidence accumulation process (arrow in top panel) is determined by all stimuli which fall under the attentional spotlight (Gaussian distribution in bottom panel). Early in the trial both the central target and foils contribute to the drift rate. Over time, the spotlight narrows on the target and the foils are suppressed. The SSP can account for typical observations on the task: more time is required to suppress the foils and resolve the conflict on incongruent trials compared with congruent trials where foil and target are the same meaning evidence accumulation process will rapidly reach the correct boundary. Fast errors are more likely on incongruent trials where the evidence accumulation process is more likely to hit the wrong boundary. Figure adapted from https://flickr.com/photos/150716232@N04/48957578602 which is available under CC licence https://creativecommons.org/licenses/by/2.0/.

The SSP is an extension of the drift diffusion framework (see Ratcliff & McKoon, 2008) for the flanker task. Like the standard diffusion model, the SSP conceptualises the decision process with each response option represented as a boundary. Following the presentation of the stimulus, a noisy process of evidence accumulation begins until a boundary is hit which triggers that response. Under the SSP, the foils contribute to the evidence accumulation process (drift rate) as well as the target, thus leading to faster evidence accumulation toward the correct response boundary on congruent trials in comparison to incongruent trials. The relative contribution of the target and foils to the drift rate is determined by the width of the attentional spotlight which continuously narrows on the target over the course of the trial. This spotlight is conceptualised as a Gaussian weight function which is centred on the target with a width at the beginning of the trial (*sd_a_*) and rate of narrowing (*rd*). The ratio of these parameters gives the foil interference time, whereby lower values indicate that foils were suppressed more effectively (White et al, 2018). Like the standard diffusion model, the distance between the response boundaries (boundary separation *A*, a measure of response caution) and non-decision-making processes (*T_er_*) parameters are also estimated. See Figure 1 for a schematic of the response selection process according to the SSP model.

#### The flanker task in autistic samples

Several studies have made use of the flanker task to investigate spatial selective attention in autism (see Table 1 for a summary).

**Table 1:** Summary of studies using the flanker task to investigate visual spatial selective attention in autism. ‘Differences overall’ indicates where there were response time or accuracy differences between the groups which were not modulated by condition. ‘Foil interference’ indicates whether response time/accuracy measures of foil interference were increased (↑), decreased (↓), or not different (=) in the autistic group relative to non-autistic group (according to studies own criterion, assumed to be ɑ < .05 if not specified).


STUDY	PARTICIPANTS	CONDITIONS	STIMULI	DIFFERENCES OVERALL	FOIL INTERFERENCE	NOTES

Adams & Jarold (2012)	15 autistic, 15 non-autistic (learning disabled) & 15 non-autistic children (age 8–17)	Congruent, incongruent	Arrow	–	↑	Manipulated target size, distance between foil and target. Autistic group performance not modulated by these manipulations.

Boland et al (2019)	36 autistic & 44 non-autistic children/adults (age 11–20)	Congruent, incongruent	Arrow (fish)	–	=	

Brandes-Aitken et al (2018)	14 autistic, 14 sensory processing dysfunction & 14 neurotypical children (age 9–11)	Congruent, incongruent	Arrow	–	=	

Brodeur et al (2018)	13 autistic & 13 non-autistic children (age 5–13)	Neutral, no foils	Symbols	Response times increased in no foil condition in autistic group	↓	Increasing delay of foils increased autistic group response times. No effect of spatial distance.

Christ et al, (2007)	18 autistic and 23 non-autistic children (age 6–15)	Congruent, incongruent, neutral	Letters, symbols	Response times and errors increased in autistic group	↑	Four possible targets, two mapped to each response button. Groups differed on age, gender and IQ

Christ et al (2011)	28 autistic and 49 non-autistic children (age 8–18)	Congruent, incongruent	Arrow (fish)	Errors increased in autistic group	↑	

Dichter & Belger (2008)	12 autistic and 22 non-autistic adults (age 18–38)	Congruent, incongruent, neutral	Arrow	Response times reduced in autistic group	↑	Trials preceded by high and low ‘arousal’ images

Dichter & Belger, (2007)	17 autistic and 15 non-autistic adults (age 20–28)	Congruent, incongruent, neutral	Arrow Eye Gaze	–	=	FMRI – congruency modulated neural response to gaze stimuli reduced in autistic group

Faja et al (2016)	19 autistic and 30 non-autistic children (age 7–11)	Congruent, incongruent	Arrow (fish)	Errors and response times increased in autistic group	↑ & ↓	ERP measured – N2 component increased in autistic group but not modulated by group

Henderson et al (2006)	24 autistic and 17 non-autistic children (age 10–12)	Congruent, incongruent	Arrow	Errors increased in autistic group	=	Presentation of stimuli and time to respond calibrated based on performance. ERP measure of error related negativity no difference in amplitude between groups

Keehn et al (2010)	20 autistic and 20 non-autistic children (age 8–19)	Congruent, incongruent, neutral	Arrow	–	=	Attention network test.

Montgomery et al (2022)	38 autistic and 50 non-autistic children (age 6–14)	Congruent, incongruent	Arrow	–	=	

Samyn et al (2014)	20 autistic, 24 ADHD & 21 neurotypical children (age 10–15)	Congruent, incongruent	Arrow	–	=	ERP measures: N2 amplitudes similar, P3, ERN similar between autistic and non-autistic groups

Sanderson & Allen (2013)	31 autistic and 28 non-autistic children (age 8–14)	Congruent, incongruent, neutral	Arrow	Response times reduced in autistic group	=	

South et al (2010)	24 autistic and 21 non-autistic children (age 8–18)	Congruent, incongruent	Arrow (foils were chevrons)	–	=	60% of trials incongruent Measured error monitoring – error related negativity reduced

Van Eylen et al (2015)	50 autistic and 50 non-autistic children (11–13)	Congruent, incongruent	Arrows	–	=	


Evidence that the suppression of foils may be less effective in autistic children comes from observations of increased response time in the incongruent condition (Christ et al, 2007), increased response time congruency effects (Christ et al, 2011) and error congruency effects (Adams & Jarold, 2012; Faja, Clarkson and Webb, 2016). On the other hand, no differences in response time congruency effects have also been observed between autistic and non-autistic children (Brandes-Aitken et al, 2018; Keehn et al, 2010; Montgomery et al, 2022; Samyn, Roeyers, Bijttebier, & Wiersema, 2017; Samyn, Wiersema, Bijttebier & Roeyers, 2014; South, Larson, Krauskop, & Clawson, 2010; Van Eylen et al, 2015), adolescent/adult (Boland, Stichter, Beversdorf and Christ, 2019) and adult (Dichter & Belger, 2007) participants. Finally, there are also studies which have revealed *reduced* response time congruency effects in autistic children (Brodeur, Stewart, Dawkins & Burack, 2018; Faja, Clarkson and Webb, 2016) and adults (Dichter & Belger, 2008).

It is clear from the above, that the flanker task presents mixed evidence as to whether the suppression of foils is less effective in autistic participants. There are methodological considerations regarding the studies reviewed above, which may partially account for the discrepancies in findings. Firstly, there is some between-study variation in how the flanker task has been employed (see Table 1) with many of those which have observed differences in interference measures deviating in working memory demands from response-stimulus mapping (Christ et al, 2007), the foil-target similarity (Brodeur et al, 2018) and stimulus presentation (Adams & Jarold, 2012; Dichter & Belger, 2008). Second, there is also variability in the dependent variable that has been used to index interference from the foils with response time congruency effects, error congruency effects, or response times on incongruent trials used as the key measure in studies describing between group differences. A further issue relating to the dependent variable is that the overall response times and/or accuracy on the task (that is, not modulated by congruency) differs between the groups in many studies (see Table 1). This suggests between group differences in response caution and/or general processing speed which would increase the error variance when measuring foil interference using response times and error rates (Hedge et al, 2018). Note also that increased intra-individual variability in response times have been observed in autistic samples (Karalunas et al, 2014) which would further inflate the error variance. Third, sample sizes in each group <= 50 which means studies are only statistically powered to observe medium to large between group differences.^1^ Existing studies have not used statistical tests which can separate absence of an effect from a lack of evidence (Lakens et al, 2020).

In the current study we addressed the methodological considerations described above. We conducted a pre-registered study using the standard version of the flanker task. Data was collected online to facilitate recruitment of a much larger sample than in previous work, increasing the precision of the between-group difference effect size estimate. We also fitted the SSP to the data to compare estimated parameters between the groups. This allowed us to combine response time and error data in a principled way, to examine which cognitive processes were involved in any differences between groups. We compared performance between autistic and non-autistic adults. There is limited evidence in adult groups, two of the three studies which have compared performance between autistic and non-autistic adults have used variations upon the standard flanker task (Brodeur et al, 2018; Dichter & Belger, 2008) and have shown *reduced* congruency effects. However, there are studies which have used visual (Remington, Swettenham, Campbell & Coleman, 2009) and crossmodal (Poole, Gowen, Warren & Poliakoff, 2015; 2018) paradigms that are analogous to the flanker task, involving interference from foils, which have produced evidence consistent with less effective suppression.

Based on the suggestion autistic people have a less effective spotlight of spatial selective attention, we predicted that a) the response time congruency effect would be increased in the autistic group compared to the non-autistic group and b) the interference time estimated from the shrinking spotlight model would be increased in the autistic group compared to the non-autistic group. We did not have any a priori expectations about group differences between the other parameters estimated using the model.

## Method

The study design and analysis were pre-registered (see https://osf.io/rmcvx & https://osf.io/3keud).^2^

### Participants

We recruited 152 autistic participants and 147 non-autistic participants to the study. See Table 2 for demographic information. Inclusion criteria were: aged 18–45, speak English (via self-report), and no learning disability (via self-report). The autistic sample had a diagnosis of autism (via self-report and as a pre-requisite for inclusion on the database, see below). The non-autistic sample had no diagnosis of autism, or other form of neurodivergence (via self-report). Participants were excluded prior to analysis who produced an overall accuracy <65% (autistic group n = 1) and for failing two attention checks on questionnaires (autistic group n = 7, non-autistic group n = 1). The final sample size in the autistic group was n = 144 and in the non-autistic group n = 146. We planned to recruit 150 to each group to account for exclusions, but slightly over and undershot each group through batch recruiting via the different platforms. A sample size of 130 in each group would give 80% power to observe a between groups difference in a one-sided between groups t-test with an effect size of d = 0.31. This was the mean effect size previously reported for increased interference effects in autistic samples (Geurts, van den Bergh & Ruzzano, 2014).

**Table 2:** Demographic information and questionnaire score of the final sample. International Cognitive Ability Resource Score (ICAR-16; Condon & Revelle, 2014) is a measure of general cognitive ability, described in detail below. Ritvo Autism and Aspergers Diagnostic Scale (RAADS-14) is an autism screening questionnaire (described below). The Cognitive Failures Questionnaire (CFQ; Broadbent, Cooper, FitzGerald & Parkes, 1982). is a measure of everyday cognitive functioning (described and analysed in the supplementary materials, S2). Detailed demographic information regarding participant sex at birth, gender, race, neurodivergence other than autism, mental health and any medications is available in S1 of the supplementary materials (see https://osf.io/w6fjm/).


GROUP	AGE (YEARS)	SEX AT BIRTH	ICAR-16	RADS-14	CFQ

Autistic	31.20 (SD = 7.23)	99 Female 43 Male 2 Intersex	7.66 (SD = 3.63)	30.20 (SD = 8.28)	57.50 (SD = 20.06)

Non-Autistic	33.60 (SD = 6.43)	94 Female 52 Male	6.95 (SD = 3.62)	10.20 (SD = 9.06)	31.42 (SD = 18.58)


#### Recruitment

Participants in the autistic group were recruited via the Simons Foundation Powering Autism Research for Knowledge (SPARK; SPARK Consortium, 2018) research match database. Recent work validating a sample from the SPARK database with electronic medical records reported an autism diagnosis in 98% of cases (Fombonne et al, 2021). Participants received a $10 voucher for taking part in the study.

Participants in the non-autistic group were recruited via Prolific (www.prolific.co). We used the Prolific recruitment tools to advertise the study to people who were living in the USA (to match the SPARK database) and were not autistic or otherwise neurodivergent. To match sex and age groupwise the study was released in batches, and we changed the recruitment criteria to target more men or women as required. Participants received an average payment of $6.67 for taking part in the study.

### Materials

All parts of the study were delivered using Gorilla (www.gorilla.sc, Anwyl-Irvine, Massonnié, Flitton, Kirkham & Evershed, 2020). Materials used in this study can be viewed here: https://app.gorilla.sc/openmaterials/802718.

#### Flanker task

The stimuli were black arrows presented on a grey background. There was a central target arrow which was flanked by four identical foils (two on the left and two on the right). The foils could be congruent (e.g. target left, foils left; 50% of trials) or incongruent with the target (e.g. target left, foils right; 50% of trials).

##### RAADS-14

The Ritvo Autism and Asperger Diagnostic Scale (RAADS-14; Eriksson, Andersen, & Bejerot, 2013) is a fourteen-item autism screening questionnaire for adults. The original study found the scale had excellent internal consistency (ɑ = .90) and that scores above 14 had a sensitivity of 97% and specificity of 46–64% for identifying autism. In the present study ɑ = .925 95%CI [.913,.935]. We included an additional question as an attention check (‘I will respond ‹true only now› to this item’).

##### ICAR-16

The International Cognitive Ability Resource (ICAR-16; Condon and Revelle, 2014) is an assessment of general cognitive ability available for online testing. There are 16 items covering Matrix Reasoning, Three-Dimensional Rotation, Verbal Reasoning and Letter and Number Series. The maximum score is 16. Total score on the ICAR-16 has convergent validity with full-scale-IQ as measured using the WAIS-IV (r = .81; Young & Keith, 2020).

### Procedure

#### Flanker Task

Participants were instructed to respond to the direction of a central target arrow, pressing the z key on their keyboard if the arrow was pointing left and m if the arrow was pointing right. Participants were instructed to respond accurately and promptly. On each trial participants were presented with a fixation cross which remained onscreen for 400, 600, 800 or 1000 ms (with equal probability of each duration across congruent and incongruent conditions). The stimuli were then presented immediately until a response was recorded. Participants completed eight blocks of 48 trials (391 trials in total). Before beginning the experiment participants completed 12 practice trials with feedback on their accuracy.

The order of testing was fixed for all participants. Participants completed demographic questions, the flanker task, the ICAR-16, the Cognitive Failures Questionnaire, and the RAADS-14 in that order. Participants were not supervised while completing the task meaning the experimenter did not monitor the participant while taking part in the study. Before beginning there was a short video of the researcher explaining the study.

### Data Analysis

Data preparation and analysis was conducted in R (version 4.1.1). We used the *tidyverse* (Wickham et al, 2019) and *janitor* (Firke, 2023) packages for data preparation, the *ggdist* (Kay, 2023) and *ggpubr* (Kassambara, 2023) packages to support data visualisation, and the *dabestr* (Ho et al, 2019) package for bootstrapping group differences, effect sizes and preparing Gardner-Altman plots.

#### Groupwise matching

**Figure 2:**
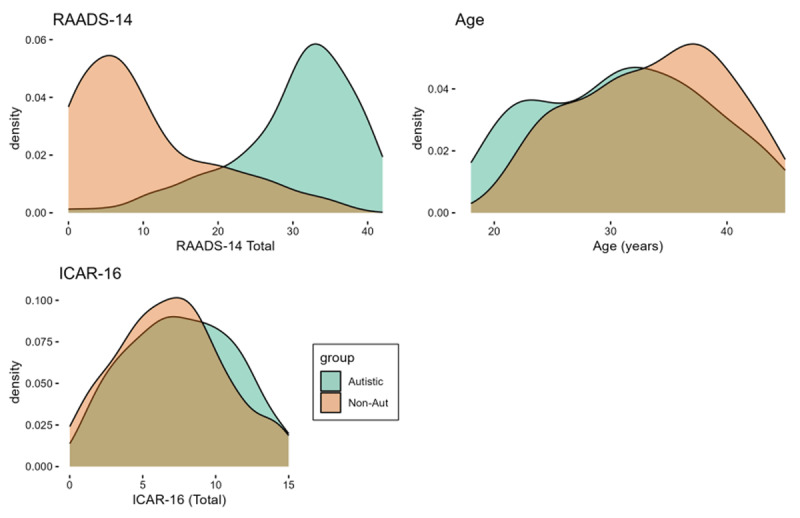
Density plots displaying autistic (green) and non-autistic (orange) group scores on the RAADS-14, age and ICAR-16. As can be seen from the figure, the groups were well matched for age and total score on the ICAR-16. As would be expected, the autistic group scored much higher on the RAADS-14 compared with the non-autistic group.

Density plots displaying the autistic and non-autistic group age, total score on the ICAR-16 (measuring general cognitive ability) and total score on the RAADS-14 (autism screening questionnaire) are displayed in Figure 2. Mean scores, effect sizes and variance ratios of the differences are given in Supplementary Materials S1.

#### Response Times

Response times <150 ms (anticipation errors; 0.28% of trials from the autistic group and 0.11% of trials from the non-autistic group) and >2000 ms (misses; 1.36% of trials from the autistic group and 0.28% of trials from the non-autistic group) were removed prior to analysis. Following response time trimming, >242 trials were included for each participant in the autistic group and >326 trials in the non-autistic group. Firstly, we calculated a congruency effect (CE) for each participant (Lakens, Scheel, & Isager, 2018). The CE was compared between the groups using an equivalence test using the *TOSTER* package^3^ (Lakens, 2017). We set the region of equivalence to d = –0.31 to d = 0.31 which was based on a meta-analytic effect size estimate of increased congruency effects on conflict tasks in autism (Geurts, van den Bergh & Ruzzano, 2014). Second, we calculated distributional plots of response time data using the *flankR* (Grange, 2016) and *dmcfun* package (Mackenzie & Dudschig, 2021) to visualise the effect of the foils over time.

#### Shrinking Spotlight Model

The Shrinking Spotlight Model (White, Ratcliff & Starns, 2011) was fitted to individual participant response times on accurate and error trials using the *flankR* package (Grange, 2016). The parameters *A, ter, p, rd* and *sd_a_* were left free to vary in the fitting routine. The fit routine searches for the closest fit between simulated and empirical cumulative distribution functions and conditional accuracy functions assessed using the Neadler-Mead algorithm to minimise the likelihood ratio chi-square statistic G^2^. Firstly 1,000 iterations of the Nelder-Mead algorithm were sampled from the default starting values recommended by Grange (2016; *A* = 0.05, *ter* = 0.3, *p* = 0.4, *rd* = 0.05, *sd_a_* = 1.5). The best fitting estimates were then passed to a second fitting procedure as starting values for 50,000 iterations. Model fits were good for both groups as measured using the Binned Bayesian Information Criterion (assessment of fits included in Supplementary Materials S4).

Interference time was calculated as *sd_a_*/rd (see White et al, 2018). Interference time was compared between the groups using a one-sided (autistic group > non-autistic group) Mann-Whitney U test. We compared the other estimated parameters; boundary separation (*A*), drift rate (*p*) and non-decision time (*ter*), using two-sided Mann-Whitney U tests. Non-parametric tests were used for comparing estimated parameters between groups as the data did not reach pre-registered criteria of normality (Shapiro Wilks test p < .05 and eyeballing QQ-plots).

## Results

Raw and aggregate data, and analysis code are available here: https://osf.io/w6fjm/.

**Figure 3:**
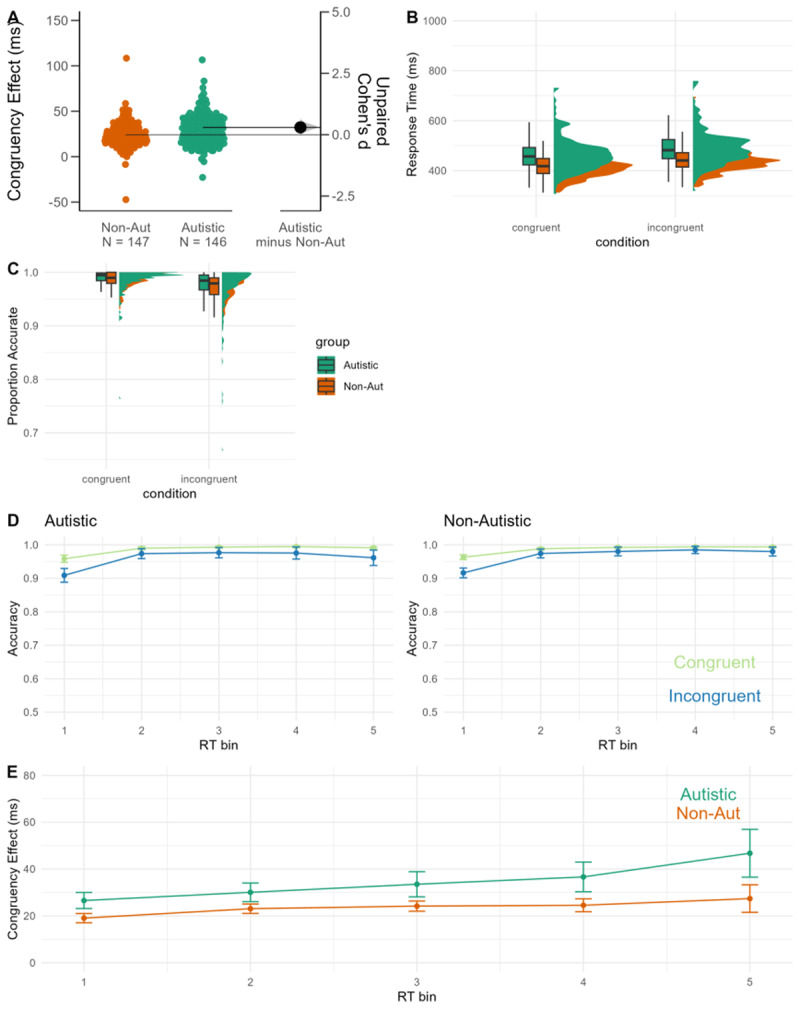
Data from the flanker task. **A:** Gardener-Altmann plot displaying the congruency effect (incongruent – congruent response times) for autistic (green) and non-autistic (orange) participants. **B:** Raincloud plot displaying the response time data **C:** raincloud plot displaying the accuracy data. **D:** Conditional Accuracy Functions for the autistic (left) and non-autistic (right) groups. **E:** Delta plot for autistic (green) and non-autistic (orange) participants. Regression models (non-pre-registered) with response time and accuracy as outcomes are reported in supplementary materials S6.

The response time congruency effect was increased in the autistic group compared to the non-autistic group (see Figure 3a). This was confirmed using an equivalence test (equivalence bound d = –0.31 to d = 0.31) which was non-significant, suggesting the estimated effect size fell outside of the equivalence bounds. The estimated effect size was significantly larger than the lower bound (t (194.42) = 5.23, *p* < .001) but did not differ from the upper boundary (t (194.42) = –0.04, *p* = .484). The null-hypothesis test was statistically significant (t (194.42) = 2.59, *p* = 0.01, mean difference = 8.07 95%CI [3.24, 16.40], d = 0.30, 95% CI = 0.07, 0.41]).

Conditional Accuracy functions are displayed in Figure 3c. Both groups show the typical effect, whereby the difference in accuracy in the congruent and incongruent condition is largest for the shortest response time bin. However, the autistic group showed an increase in the error congruency effect in the longest response time bin. Similarly, the delta plots (see Figure 3d) which show the response time difference between the incongruent and congruent conditions by deciles are flat for the non-autistic group but are positive going for the autistic group with a large increase for the longest response time bin.

## Shrinking Spotlight Model

**Figure 4:**
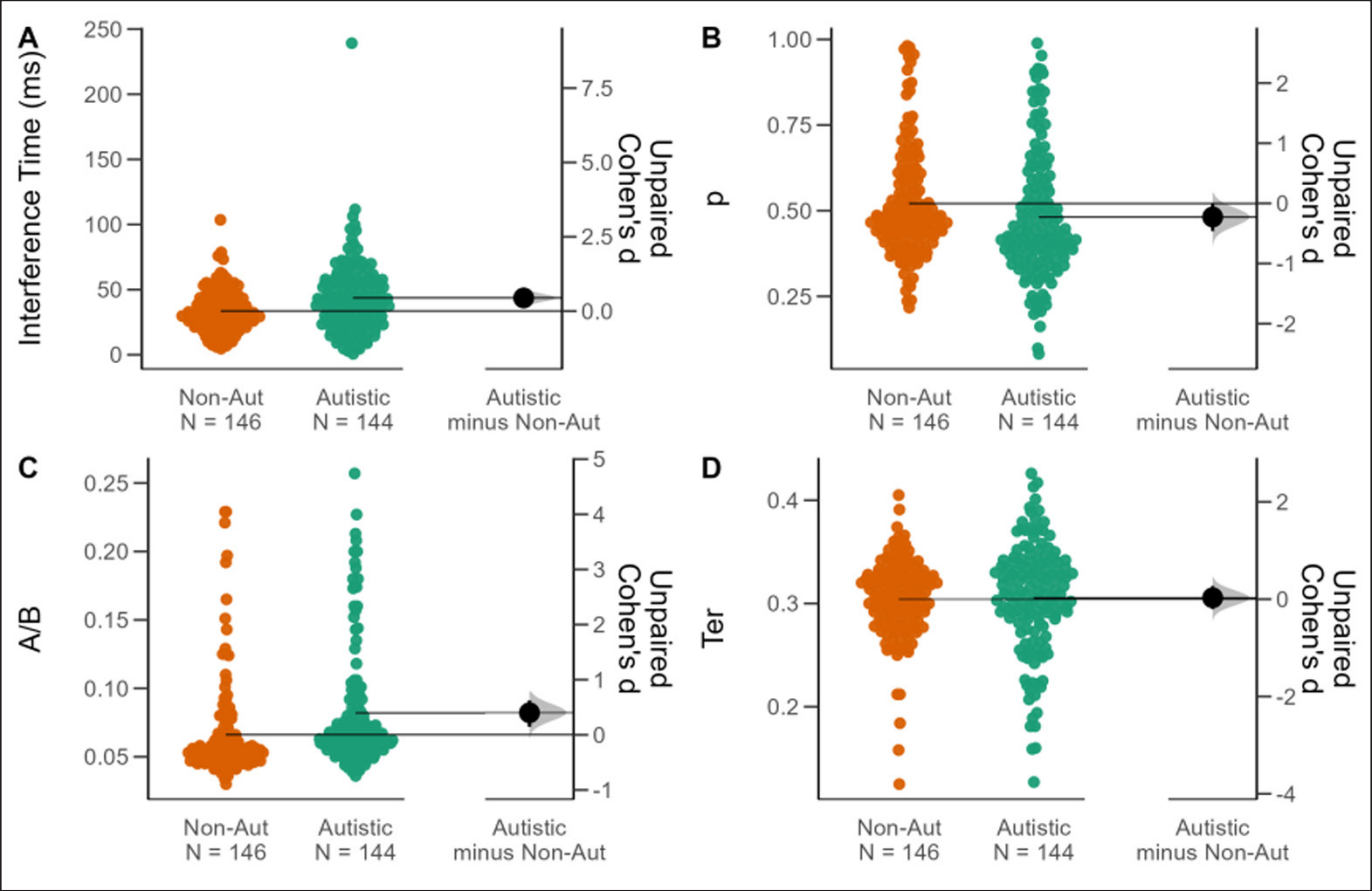
Gardner-Altmann plot displaying the the autistic group (green) and non-autistic group (orange) parameters estimated using the Shrinking Spotlight Model **A:** interference time (sd/rd) **B:** perceptual sensitivity (*p*) **C:** Boundary separation (*A/B*) **D:** non-decision time (*T_er_*).

Estimates of interference time are displayed in Figure 4a. Interference time was increased in the autistic group (mean = 43.61, SD = 28.04) compared with the non-autistic group (mean = 33.50, SD = 15.99). This was confirmed using a Mann Whitney U test (W = 13008, *p* < .001, mean difference = 10.10, 95%CI [5.24, 16], d = 0.44, 95% CI [0.23, 0.64]).

The other parameters which were estimated from the Shrinking Spotlight Model (perceptual sensitivity *p*, boundary separation *A/B* and non-decision time *T_er_*) are also displayed in Figure 4. The parameter *p* was reduced, and *A/B* was increased in the autistic relative to the non-autistic group, whereas *T_er_* was not different between the groups. This was confirmed using a series of Mann-Whitney U tests (reported in Table 3).

**Table 3:** Descriptive and test statistics for comparison of parameter estimates perceptual sensitivity (p), boundary separation (A/B) and non-decision time (Ter) for the Autistic (A) and Non-Autistic (NA) groups.


PARAM	A MEAN (SD)	NA MEAN (SD)	U	*p*	MEAN DIFFERENCE [CI]	d [CI]

*p*	0.48 (0.19)	0.52 (0.16)	8577.50	.006	–0.04 [–0.07, <.001]	–0.23 [–0.46, <.01]

*A/B*	.082(.044)	.066 (.036)	14420	<.001	016 [.006,.026]	0.40 [0.15, 0.62]

*Ter*	0.31 (0.05)	0.30 (0.04)	11129	.388	<.01 [–.01,.01]	0.03 [–0.20, 0.27]


## Discussion

In the present study autistic and non-autistic adults completed an online version of the flanker task. In line with our predictions, a) congruency effects were increased in the autistic group, and b) interference time estimated using the SSP was increased in the autistic group.

As detailed in the introduction there is previous evidence that response time congruency effects are increased on a standard version of the flanker task in autistic children (e.g. Christ et al, 2011), but the evidence base is mixed (e.g. Boland et al, 2019). Notably, the effect size of the between-group difference in congruency effects reported here (d = 0.30) was very similar to the average described across interference studies (g = 0.31, Geurts, van den Bergh & Ruzzano, 2014), with the current study testing adults and using a larger sample size.

We extended previous work using distributional plots to look at the time course of response times and accuracy. Both groups showed the typical effect in conditional accuracy functions (i.e., the error congruency effect was largest in the fastest response time bin). However, the autistic group also showed a slight increase in the congruency effect for the slowest response time bin. Furthermore, the delta plots were more positive for the autistic group, with an increase in the slowest response time bin. This might suggest that there was early activation from the foils for the non-autistic group, which was effectively suppressed later in the trial. The autistic group showed this early activation, but may have not completely suppressed the foil, with at least a partial activation persisting which impacted on performance in slower response times (see Burle, Spieser, Servant & Hasbroucq, 2014). This speculation is supported by the modelling of the decision-making process. We fitted the Shrinking Spotlight Model (White et al., 2011) to individual participant response time and accuracy data. The estimated interference time was increased in the autistic group in comparison to the non-autistic group. According to the Shrinking Spotlight Model this would be consistent with the suggestion that the spotlight of spatial selective attention functions less effectively in autism (Burack, 1994). The spotlight was broader for longer meaning that the foils had more impact on evidence accumulation during response selection. This has provided a valuable insight into the nature of selective attention in autism. Previous work has shown that autistic adults show more foil interference under conditions of high load (Remington et al, 2009) and in a visual-tactile task where the target was presented at threshold level (i.e., making the task difficult. Poole et al, 2015, 2018). The present study has indicated that increased interference from foils is observed on a visual task under low-load, low-difficulty conditions. It would be valuable in future work to systematically test foil interference across load and difficulty conditions to constrain theories of selective attention in autism.

In the current study, fitting participant data to the shrinking spotlight model revealed that the estimated boundary separation (*A*) was increased, and perceptual processing (*p*) was reduced in the autistic group. This indicates that non-interference parameters contributed to the differences in congruency effects and as such caution should be taken when interpreting increased response time or error congruency effect as a measure of foil interference. Increased boundary separation in autistic participants has previously been observed (Karalunas et al, 2018; Pirrone et al, 2017; Pirrone et al, 2020; although see Poole, Miles, Gowen & Poliakoff, 2021; Manning et al, 2022), as has a slower and more deliberate decision-making style (Brosnan, Lewton & Ashwin, 2016; Brosnan & Ashwin, 2022).^4^ It would be useful to establish whether increased boundary separation (i.e., prioritising accuracy over speed) is a robust feature of decision making in autism, to better characterise autistic cognition and decision making.

A constraint in the interpretation of the current findings is that a large proportion of the autistic group identified themselves as having ADHD (46% of the sample; see supplementary materials). As distractor interference has also been observed in people with ADHD (Onandia-Hinchado, Pardo-Palenzuela & Diaz-Orueta, 2021), we repeated the analysis with participants with an ADHD diagnosis excluded and the pattern of results remained the same (see Supplementary materials S7). Nonetheless, it is also likely that further participants in the autistic group could have undiagnosed ADHD. One approach for future studies would be to take a measure of ADHD traits (e.g. Kessler et al, 2005) to determine whether these traits moderate differences in foil interference. An alternative would be to adopt a transdiagnostic approach (Astle, Holmes, Kievit & Gathercole, 2022), recruiting a neurodiverse sample to complete selective attention tasks alongside measures of challenges and strengths using a data driven approach to better characterise inefficient suppression. Additionally, a large proportion of participants in the current study were female. In non-autistic populations there is evidence that foil interference is increased in women (Stoet, 2010), so it would be valuable to include sex in future transdiagnostic clustering approaches.

## Conclusion

This study has generated findings consistent with an inefficient spotlight of spatial selective attention in autism. Autistic adults produced an increased congruency effect on a standard version of the flanker task compared with non-autistic adults. Fitting participant data to a model of spatial selective attention revealed that interference time was increased in the autistic group. It is also important to note that there were between group differences in response conservativeness and efficiency of processing. This highlights the value of modelling decision making processes when trying to understand differences in autistic cognition.

## Data Accessibility Statement

Raw, aggregated data and analysis code can be found here: https://osf.io/w6fjm/.

## Footnotes

Power analysis conducted using *pwr* package in R (Champley, 2013) indicated that n = 50 in each group is powered to observe an effect size of d = 0.57 using a two-sided, independent sample t-test with α = .05 and *β* = .80.

The pre-registered analysis of the post-error slowing and cognitive failures questionnaire (Broadbent et al, 1982) are included in the supplementary materials.

TOSTER also provides a t-test comparing the CE between the groups which we report here for completeness, although this analysis was not pre-registered.

Relatedly, in analysis reported in section S5 of the supplementary materials we observed *increased* post-error slowing in the autistic relative to the non-autistic group.
